# Measurement of the QT interval using the Apple Watch

**DOI:** 10.1038/s41598-021-89199-z

**Published:** 2021-05-24

**Authors:** Carmen Anna Maria Spaccarotella, Serena Migliarino, Annalisa Mongiardo, Jolanda Sabatino, Giuseppe Santarpia, Salvatore De Rosa, Antonio Curcio, Ciro Indolfi

**Affiliations:** 1 Division of Cardiology, University Magna Graecia, 88100 Catanzaro, Italy; 2 Mediterranea Cardiocentro, 80122 Naples, Italy

**Keywords:** Cardiology, Health care

## Abstract

The inherited and acquired long QT is a risk marker for potential serious cardiac arrhythmias and sudden cardiac death. Smartwatches are becoming more popular and are increasingly used for monitoring human health. The present study aimed to assess the feasibility and reliability of evaluating the QT interval in lead I, lead II, and V2 lead using a commercially available Apple Watch. One hundred nineteen patients admitted to our Cardiology Division were studied. I, II, and V2 leads were obtained after recording a standard 12-lead ECG. Lead I was recorded with the smartwatch on the left wrist and the right index finger on the crown. Lead II was obtained with the smartwatch on the left lower abdomen and the right index finger on the crown. The V2 lead was recorded with the smartwatch in the fourth intercostal space left parasternal with the right index finger on the crown. There was agreement among the QT intervals of I, II, and V2 leads and the QT mean using the smartwatch and the standard ECG with Spearman’s correlations of 0.886; 0.881; 0.793; and 0.914 (p < 0.001), respectively. The reliability of the QTc measurements between standard and smartwatch ECG was also demonstrated with a Bland–Altman analysis using different formulas. These data show that a smartwatch can feasibly and reliably assess QT interval. These results could have an important clinical impact when frequent QT interval monitoring is required.

## Introduction

The QT interval is measured from the beginning of QRS to the end of the T-wave. It expresses ventricular depolarization and repolarization. The importance of measuring the QT interval is related to the fact that, if prolonged, it may cause dangerous arrhythmias or even sudden cardiac death^1^. A long QT interval can be congenital or acquired. Congenital causes of long-QT syndrome are linked to genetic mutations. There is an estimated case prevalence of 1 in 2000 in the general population with mutation found in about 75–80% of cases^2,3^. On the other hand, acquired causes of long QT are frequently related to the use of drugs that, alone or in combination, can cause potentially dangerous QT interval prolongation, especially when the long QT is accompanied by diarrhea and/or hypokalemia^4^.

Nowadays, numerous drugs can pathologically prolong the QT interval, exposing patients to the risk of Torsade-de-Pointes (TdP). Current classifications divide drugs into four different risk classes: drugs that have a known, possible, or conditional risk of TdP, and drugs to avoid in patients diagnosed with long-QT syndrome^5^.

Currently, smartwatches are becoming particularly popular to enhance health monitoring and care delivery. The Apple Watch, for instance, can perform an electrocardiogram using a single peripheral lead (lead I), which is obtained through a circuit between the detector on the back of the watch and the digital crown. Apple Watch has received FDA approval for atrial fibrillation detection. Recently, we have shown that it is possible to obtain nine-lead ECGs by moving the Apple Watch on the body to detect ST changes, similar to obtaining a standard ECG^6^.

The aim of this study was to compare the feasibility and reliability of using the Apple Watch to calculate a QT interval to those of using a standard ECG to calculate a QT interval.

## Experimental section

### Material and methods

The Ethical Committee of the University Magna Graecia approved the study (project identification code: 417), and all subjects included in this study gave written informed consent; participants did not receive financial compensation. This study followed the relevant guidelines and regulations for case series.

The study population included 100 patients admitted to the Cardiology Division of the University Magna Graecia and 19 healthy subjects used as a control. Characteristics of all subjects studied and possible risk factors for QT prolongation are reported in Table 1. A researcher (SM) recorded the ECGs with the smartwatch. All ECGs were blinded and analyzed by two experienced cardiologists (SM and CS). In all patients, the standard ECG was recorded upon admission to the Cardiology Division. Of the total of 55 patients admitted to our CCU with the diagnosis of acute coronary syndromes (ACS), the patients with ST-Elevation Myocardial Infarction (STEMI) were admitted directly to the Cath lab and the ECGs were acquired in the Cath lab during the preparation of the patient (positioning electrodes, disinfection, recording of name, etc.), before the recanalization. Differently, the patients with non- ST-Elevation Myocardial Infarction (NSTEMI) were admitted to CCU and, in the same way, both ECGs (standard and with the apple watch) were acquired at the same time.

**Table 1:** Characteristics of the study subjects.

Variable	ACS (*n* = 55)	NO_ACS (*n* = 45)	Control (*n* = 19)	*p*-value
Age, y ± SD	63 ± 11	52 ± 21	33 ± 11	0.0027*
				< 0.0001^
				0.0001°
Male, *n* (%)	46 (39)	24 (20)	6 (5)	0.0025*
				0.0002^
				0.4225°
Hypertension, *n* (%)	43 (36)	26 (22)	2 (2)	0.1181*
				< 0.0001^
				0.0014°
Diabetes, *n* (%)	15 (13)	9 (8)	0 (0)	> 0.9999*
				0.1296^
				0.5638°
Dyslipidemia, *n* (%)	45 (38)	17 (14)	0 (0)	< 0.0001*
				< 0.0001^
				0.0177°
Smokers, *n* (%)	17 (14)	4 (3)	0 (0)	0.0126*
				0.0072^
				> 0.9999
Prior MI, *n* (%)	8 (7)	6 (5)	0 (0)	> 0.9999*
				0.2734^
				0.3960°
Prior stroke/TIA, *n* (%)	2 (2)	0 (0)	0 (0)	0.4833*
				0.8694^
				> 0.9999°
Obesity, *n* (%)	4 (3)	1 (1)	0 (0)	0.6371*
				0.5248^
				> 0.9999°
Hypokalemia, n (%)	1(2)	0(0)	0(0)	0.9713*
				> 0.9999^
				> 0.9999°
Antiarrhythmic drugs, n (%)	2(4)	0(0)	0(0)	0.4833*
				> 0.8694^
				> 0.9999°
Antidepressant/antipsychotic drugs, n (%)	2(4)	2(4)	0(0)	> 0.9999*
				> 0.9999^
				> 0.9999°
Antibiotic/antifungal/antiviral drugs, n (%)	2 (4)	1(2)	0(0)	> 0.9999*
				> 0.9999^
				> 0.9999°

*ACS* acute coronary syndrome, *MI* myocardial infarction, *TIA* transient ischemic attack.

p-value is to be interpreted as follows: *ACS vs NO_ACS; ^ACS vs CONTROL; °NO_ACS vs CONTROL. A p-value of < 0.05 was considered statistically significant.

#### Smartwatch ECG acquisition

**Figure 1:**
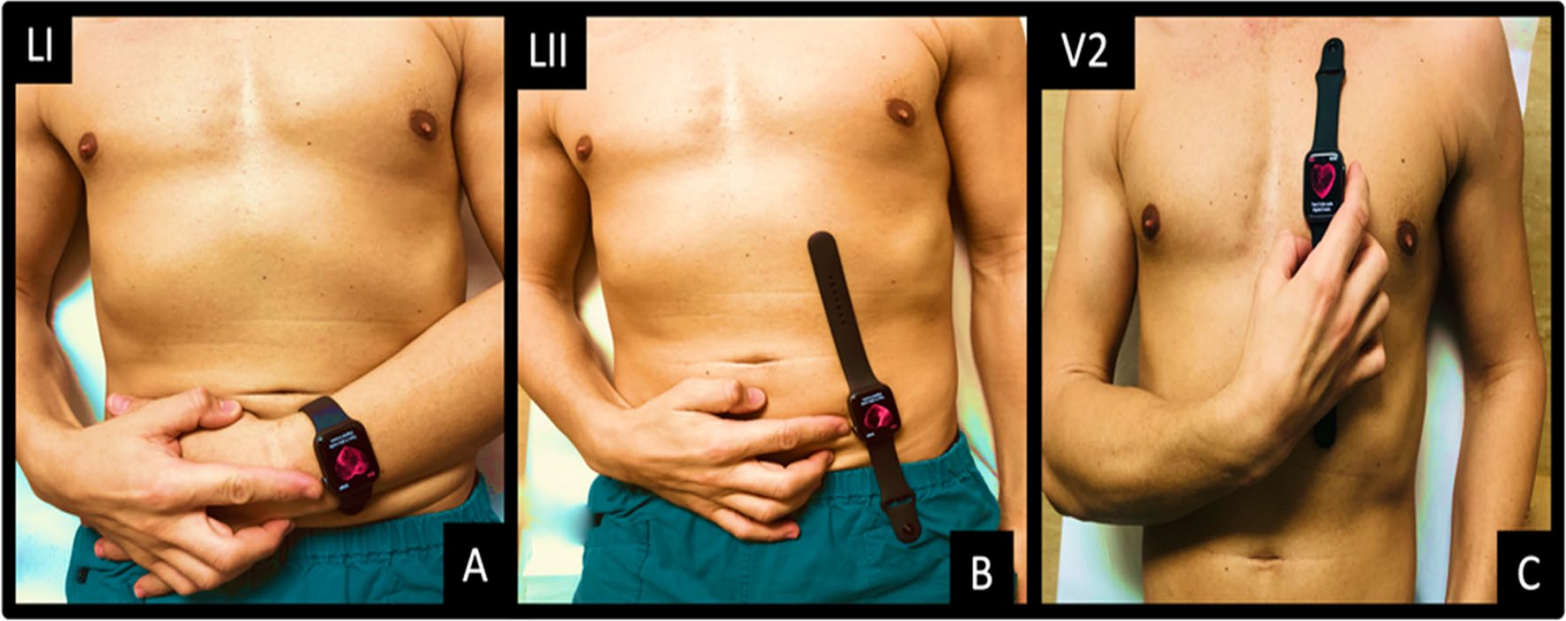
Method used to collect lead I, lead II, and the V2 lead with the smartwatch. Lead I was recorded with the smartwatch on the left wrist and the right index finger on the crown (**A**). Lead II was obtained with the smartwatch on the left lower abdomen and the right index finger on the crown (**B**). Chest lead V2 was recorded with smartwatch in the fourth intercostal space left parasternal with the right index finger on the crown (**C**).

Lead I, lead II, and V2 lead tracings were obtained using a commercially available Apple Watch Series 4 (Apple Inc., Cupertino, CA, USA) immediately after the recording of the standard 12-lead ECG. All ECG leads were obtained by creating a circuit between the back of the watch resting on the specific body surface of interest and the right index resting on the crown for 30’s. Figure 1 reports in detail the method used to acquire the smartwatch multichannel ECG.

Lead I (I) was recorded with the smartwatch on the left wrist and right index finger on the crown (Fig. 1A). Lead II (II) was obtained with the smartwatch on the left lower abdomen and right index finger on the crown (Fig. 1B). Chest lead V2 was recorded with the smartwatch in the fourth intercostal space left parasternal with the right index finger on the crown (Fig. 1C). All recorded 30’s ECGs were digitally stored using the health application of iPhone Series 10 in pdf format. The measurements of the QT interval were made on the printout of the ECG in pdf format. The advantage of saving ECGs in pdf format is that it can be sent also via e-mail.

#### Standard ECG acquisition

The standard 12-lead ECGs were performed using commercially available electrocardiography (PHILIPS, 3000 Minuteman Road Andover; MA 01810, USA) with a paper speed of 25 mm/s.

#### QT interval measurements

The QT interval was measured from the beginning of the QRS complex to the end of the T-wave, as an average of three beats. Additionally, to minimize the error in detecting the true end of the T wave and the correct QT interval we used a tangent drawn to the steepest last limb of the presumed T-wave to define the end of the T-wave as the intersection of this tangent with the baseline^7^. Also, in the case of the presence of U wave, we not included it in the measurement of QT interval because there are no accepted reference values for the QU interval, and often dedicated electrocardiographic techniques are needed before one can focus on the U-wave^7^. Additionally, in the case of bundle branch block, we measured from the beginning of the QRS complex to the end of the T wave, using the same calculation method used in the absence of bundle branch block.

Adjusting the QT interval for heart rate (QTc) was done according to Bazett’s formula (QTcB). QTcB = QT/RR1/2^8^. QTcB is equal to the QT interval in milliseconds divided by the square root of the RR interval in milliseconds. When the heart rate is particularly fast or slow, Bazett’s formula may not be accurate. Therefore, we also calculated the QTc using Framingham’s (QTcFra = QT + 0.154 (1 − RR))^9^ and Fridericia’s (QTcFri = QT/RR1/3) formulas^10^.

QTc was measured in I, II, and V2 leads both individually and as an average of all three leads (QT mean) with Bazett’s, Fidericia’s, and Framingham’s formulas.

### Statistical analysis

One-way factorial analysis of variance (ANOVA) and non-parametric Kruskal–Wallis test was used in comparing the three groups to analyze differences of variables. A p-value of < 0.05 was considered statistically significant.

The Shapiro–Wilk test was used to assess the normality of continuous variables. Correlation between two technologies was assessed using linear regression and estimated with Pearson’s analysis for normally distributed data and Spearman’s analysis for nonparametric data. In detail, we compared agreement between Smartwatch and Standard ECG for QT interval and corrected QT interval for heart rate.

The results were interpreted as follows: weak agreement = less than 0.30; regular agreement = 0.30–0.60; strong agreement = 0.60–0.90; very strong agreement = 0.90–0.10.

A plot of differences between QT and QTc interval measured using the standard and smartwatch ECG was done according to the method described by J.M. Bland and D.G. Altmann^11^.

Furthermore, assuming the results of standard ECGs as the reference, we analyzed the sensitivity and specificity of Smartwatch ECG to identify prolonged QTc interval (QTc > 460 ms).

Statistical analysis was performed using IBM SPSS (v26, IBM Corp., Armonk, NY, USA).

## Results

A total of 119 subjects in sinus rhythm were enrolled in this study, 55 (46%) with ACS, 45 (38%) without ACS, and 19 (16%) healthy controls. The mean age of the subjects included in the study was 55 ± 23 years and 58% were male. The clinical characteristics of the subjects included in this study are described in Table 1.

The Shapiro–Wilk test was used to assess the normality of continuous variables, which resulted in a normal distribution only for the QTc of lead I (QTc-I) interval using Bazett’s formula and the QTc of lead II (QTc-II) interval using Friedericia’s formula. Accordingly, the correlation between QTc using the smartwatch (QTc-a) and QTc using the standard ECG (QTc-e) was estimated with Spearman’s analysis for non-parametric data.

There was a strong agreement between the QT-I, QT-II, QT-V2, and QT-mean intervals of smartwatch and standard electrocardiography. Respectively, the Spearman’s correlation coefficients were: 0.886; 0.881; 0.793; and 0.914 (p < 0.001). Finally, a strong correlation was found between the HR of smartwatch electrocardiography tracings and standard electrocardiographs (Spearman’s correlation coefficient: 0.893 (p < 0.001)).

**Figure 2:**
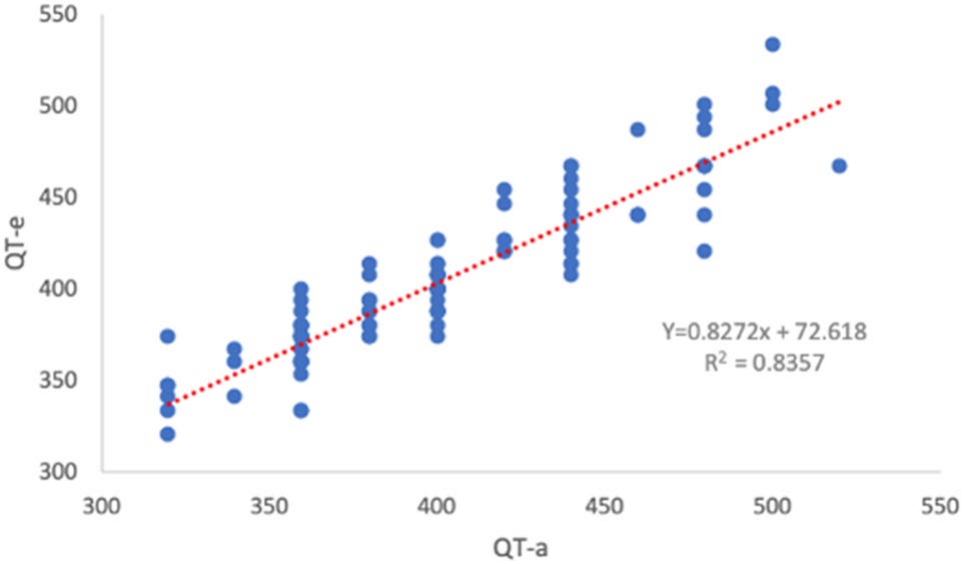
Regression analysis: correlation of mean QT-a (ms) and mean QT-e (ms). Scatterplot and fitted line showing the linear association between the QT mean interval (measured as an average of lead I, lead II, the V2 lead) performed using a smartwatch (QT-a) and standard (QT-e) ECG.

Figure 2 shows the regression analysis of the mean QT interval obtained from the smartwatch ECG and the mean QT interval recorded with the standard ECG. In the Supplementary Table S1 we showed the exact value of mean QT interval using standard and apple ECG traces.

**Figure 3:**
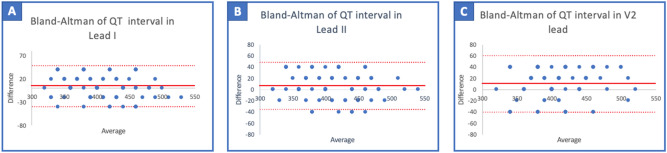
Comparison of QT measured with the standard ECG and the smartwatch ECG. Bland–Altman plot indicating the level of agreement between the smartwatch ECG and the standard 12-lead ECG measurement of the QT (ms) interval in lead I (**A**), lead II (**B**), and the V2 lead (**C**). The solid red line represents the bias and dashed red lines the upper and lower limit of agreement (LOA).

Additionally, a good concordance was found between the QT measured with the smartwatch ECG and the standard ECG. Using the Bland–Altman analysis, we found a bias of 5 ms (95% limit of agreement (LoA) − 40 to + 49 ms) with lead I; 6 ms (95% LoA − 35 to + 48 ms) with lead II; and 10 ms (95% LoA − 40 to + 60 ms) with the V2 lead (Fig. 3).

In the possibility of inaccuracy in the calculation of the QT interval in urgent situations (artifacts of the trace due to movement of the patient) and a faster modification of this interval (electrolyte changes) during the early phase of acute coronary syndrome we have reanalyzed the data also excluding patients diagnosed with STEMI (Supplementary Fig. S1).

At the same way, a good concordance was found between the QT measured with the smartwatch ECG and the standard ECG.

Using the Bland–Altman analysis, we found a bias of 4 ms (95% limit of agreement (LoA) − 41 to + 50 ms) with lead I; 6 ms (95% LoA − 35 to + 48 ms) with lead II; and 10 ms (95% LoA − 40 to + 60 ms) with the V2 lead (Supplementary Fig. S1).

The regression analysis of HR and the QT interval in the I, II, and V2 leads using a smartwatch ECG and a standard ECG are reported in Supplemental Figs. S2–S5. Additionally, we have corrected the QT interval for RR (QTc) using Bazett’s, Friederica’s, and Framingham’s formula. Testing the correlation between the QTc interval in lead I, lead II, the V2 lead, and the mean QTc of the smartwatch and standard ECGs using Bazett’s formula, we found Spearman’s correlation coefficients of 0.800; 0.775; 0.688; and 0.816, respectively (p < 0.001).

Additionally, we performed a correlation analysis of the QTc interval in lead I, lead II, the V2 lead, and the mean QTc of the smartwatch and standard ECGs using Friedericia’s formula. We found Spearman’s correlation coefficients of 0.813; 0.781; 0.674; and 0.817, respectively (p < 0.001). We also performed the same analysis of the QTc interval in lead I, lead II, the V2 lead, and the mean QTc of the smartwatch and standard ECGs using Framingham’s formula. We found Spearman’s correlation coefficients of 0.886; 0.880; 0.793; and 0.913, respectively (p < 0.001).

Additionally, we performed a Bland–Altman analysis of QTc using Bazett’s Formula, revealing a bias of 9 ms (95% LoA − 44 to + 63 ms) with lead I; 11 ms (95% LoA − 44 to + 67 ms) with lead II; 15 ms (95% LoA − 44 to + 73 ms) with the V2 lead; and 12 ms (95% LoA − 32 to + 55 ms) with the mean QTc.

In the supplementary section we also reported the Bland–Altman of QTc mean with Bazzet’s excluding patients with STEMI, revealing a bias of 9 ms (95% LoA − 45 to + 63 ms) with lead I; 11 ms (95% LoA − 42 to + 65 ms) with lead II; 14 ms (95% LoA − 46 to + 76 ms) with the V2 lead; and 12 ms (95% LoA − 32 to + 55 ms) with the mean QTc (Supplementary Fig. S7).

Additionally, we performed a Bland–Altman analysis of QTc using Friedericia’s and Framingham’s formulas. We revealed a bias of 7 ms (95% LoA − 40 to + 55 ms) with lead I; 10 ms (95% LoA − 38 to + 57 ms) with lead II; 13 ms (95% LoA − 40 to + 66 ms) with the V2 lead; and 10 ms (95% LoA − 26 to + 46 ms) with the mean QTc interval using Friedericia’s formula and a bias of 5 ms (95% LoA − 39 to + 49 ms) with lead I, 7 ms with lead II (95% LoA − 35 to + 48 ms), 10 ms (95% LoA − 39 to + 60 ms) with the V2 lead, and 7 ms (95% LoA − 25 to + 39 ms) with the mean QTc using Framingham’s formula.

**Figure 4:**
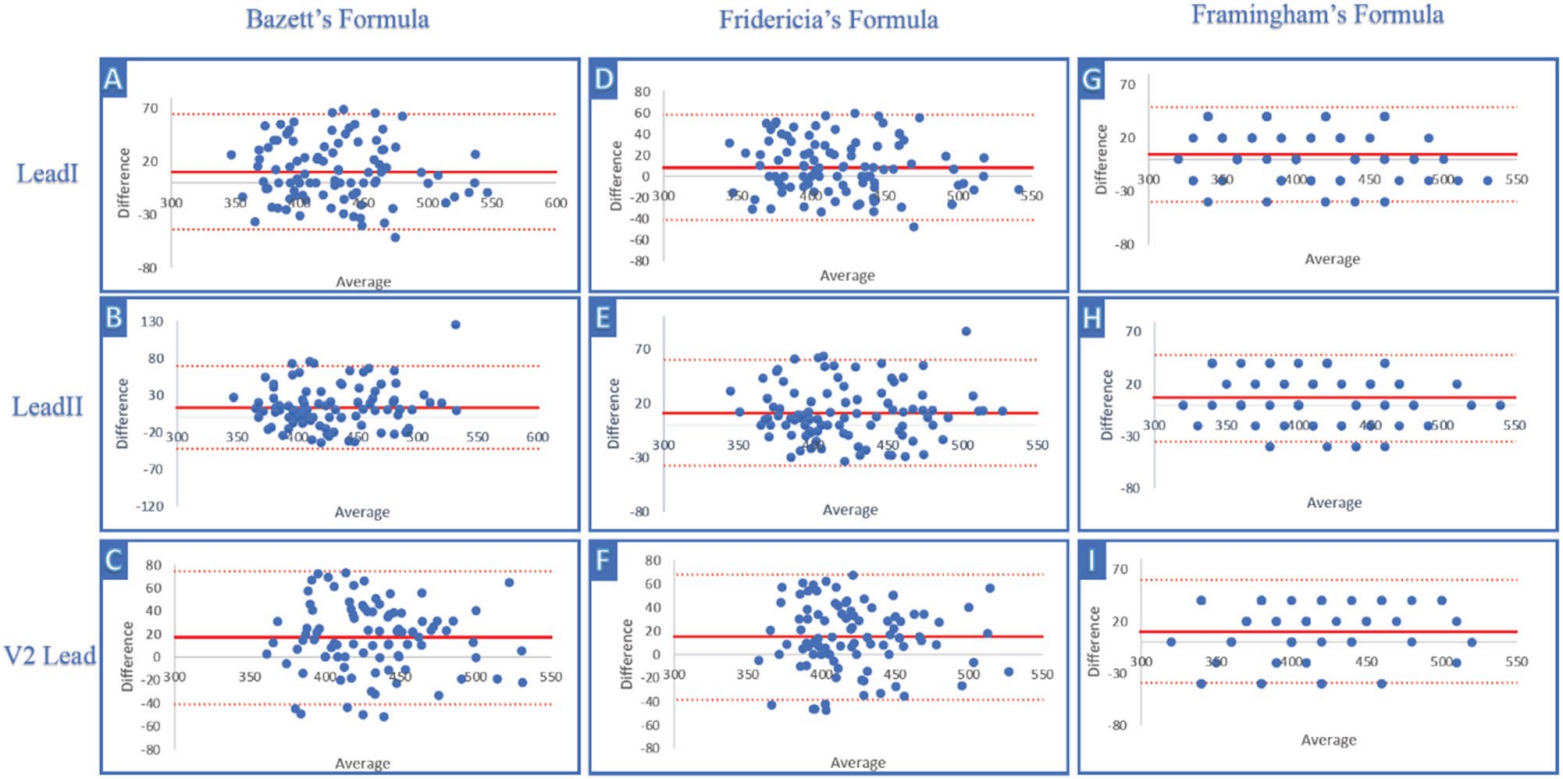
Comparison of QTc mean using Bazett’s, Friedericia’s, and Framingham’s formulas between standard and smartwatch ECG. Bland–Altman plot indicating the level of agreement between the smartwatch ECG and standard 12-lead ECG measurement of QTc (ms) using Bazett’s formula (three panels on the left: (**A**–**C**)); Friedericia’s formula (three panels in the center: (**D**–**F**)); and Framingham’s formula (three panels on the right: (**G**–**I**)), respectively, from the top to the bottom: lead I, lead II, and the V2 lead. The solid red line represents the bias and dashed red lines the upper and lower LOA.

Figure 4 shows the Bland–Altman plots of QTc measured with Bazzet’s, Framingham’s and Fidericia’s formulae.

Concordance in detecting a prolonged QTc interval (> 460 ms) of the two technologies was assessed using Cohen’s kappa coefficient. The results of Cohen’s kappa were interpreted as follows: poor agreement = less than 0.20; fair agreement = 0.20–0.40; moderate agreement = 0.40–0.60; good agreement = 0.60–0.80; and very good agreement = 0.80–1.00.

A good agreement was found between the smartwatch and standard ECG for the identification of a prolonged QTc interval using Bazett’s formula (QTc mean): Cohen’s kappa 0.73 (95% CI 0.67 to 0.79). Assuming the results of standard ECGs as the reference, the sensitivity and specificity in detecting a prolonged QTc interval was 69% (95% CI 0.61 to 0.75) and 88% (95% CI 0.85 to 0.91), respectively.

To measure interobserver variability, QT interval measurements in lead I, lead II, and the V2 lead and RR interval measurements were repeated by a second blinded cardiologist and the intraclass correlation coefficient (ICC) was calculated using Cohen’s Kappa coefficient. A good agreement was found: Cohen’s kappa 0.619 (95% CI 0.57 to 0.67).

A representative example of a pathological QT interval is reported in Supplemental Fig. S6. Three patients were excluded from the study. One was excluded due to low smartwatch signal quality and alterations in the T wave, which made it impossible to calculate the QT interval due to the lack of patient cooperation. Two other patients were excluded due to atrial fibrillation.

## Discussion

The major finding of the present study is that the Apple Watch can accurately measure the QT interval compared with standard ECG. These data could be of great interest to the wide diffusion of smartwatches when there is a need for frequent checking of the QT interval at home.

It has previously been shown that the QT interval is different in different leads^7^. The QT interval should be classically measured in lead II^7^, which cannot be measured with the Apple Watch on the right wrist. In addition, sometimes the T wave is not easily recognizable in lead II; therefore, an alternative precordial lead (V2 or V6) should be used.

A previous study by our group showed that by appropriately moving the smart-watch in different body positions it is also possible to obtain lead II and six thoracic leads^6^. Therefore, in our study, we calculated the QT interval with the Apple Watch in lead I, lead II, and the V2 lead.

In this study, we have chosen to examine the QT interval of the ECG because its monitoring could be helpful in particular clinical situations that could expose patients to a prolongation of the QT interval, increasing the risk of life-threatening arrhythmias.

The data show that the QT measurement in the three leads explored using the Apple Watch is comparable to that obtained using the standard ECG both in terms of QT and QTc corrected with Bazett’s, Fridericia’s, and Framingham’s formulas. In particular, we obtained the best correlation indices between the two methods using the first derivation, which is also the one obtained by leaving the watch in its routine position. This data could be of interest as it increases the patient’s compliance and reduces the possible errors that the patient can make by moving the watch to the different body positions that are re-quired to obtain additional leads. This operation could be useful only in those cases in which the T wave is not well defined in the first derivation, making it difficult to calculate the QT intervals. The lower correlation between the two methods in monitoring the QT interval in lead II and the V2 lead could be interpreted as a procedural error by the patient in positioning the Apple Watch, which implies small changes in the morphology of the ECG trace that inevitably affect the QT interval.

A recent study using a smartwatch demonstrated similar results^12^. However, in this latter study, the lead II was obtained by positioning the watch on the left ankle, which could be difficult to obtain in severely obese subjects or subjects with impaired function of the left limb. In contrast, in our study, we obtained lead II with the smartwatch on the left lower abdomen and the right index finger on the crown. Additionally, we calculated the QT in the V2 lead, whereas in the study by Strik et al. the QT was calculated in the V6 lead^12^.

Today, however, it is not possible to perform an automatic measurement of the QT interval with the Apple Watch (while an algorithm for the automatic diagnosis of atrial fibrillation is available). Therefore, in our study, the ECG obtained with the smartwatch was printed and the QT was manually calculated. In this regard, an automatic system that allows for the measurement of the QT segment, possibly using artificial intelligence, is desirable.

It is known that the heart rate affects the QT interval. Decreasing the heart rate in-creases the QT interval and the opposite occurs with tachycardia. So, a correction of the QT interval is necessary depending on the heart rate. Henry Bazett was the first to correct the QT interval with the HR in 1920 by introducing QTc^8^, which is widely used in the clinical setting. However, we also tested the reliability of QT measured with the smartwatch corrected by Fridericia’s and Framingham’s formulae, which may be more accurate in specific heart rate ranges^8,9^.

The remote monitoring of the QT interval could be of importance in different settings. The major application of the finding of the present study could be the use of the smartwatch for frequent monitoring of QT interval after drug therapies with or without specific conditions (diarrhea, electrolyte abnormalities, hypoxia) that may affect this QT interval. For instance, many drugs, such as haloperidol, tricyclic antidepressants, droperidol, thioridazine, quetiapine, clozapine, amiodarone, furosemide, domperidone, quinidine, sotalol, procainamide, ranolazine, and azithromycin, may be associated with QT prolongation^13^. Some anticancer drugs, such as anthracyclines, can also have proarrhythmic effects, including prolongation of the QT interval^14^.

A prolonged QT interval is also described after myocardial infarction and might be related to viable myocardium or systemic inflammation^15,16^.

Bessière et al. showed that in patients in the intensive care unit, excessive QT prolongation was observed in 36%, particularly in patients treated with hydrochlorichin alone or in combination with azithromycin^17,18^. A recent consensus recommends stopping this drug when the QTc exceeds 500 ms or when the QTc is prolonged for over 60 ms compared to baseline, or ventricular ectopy appears^19^.

QT interval monitoring could be necessary for patients with acute allograft rejection after heart transplantation where an increased QT interval on the electrocardiogram is observed^20^; additionally, QT interval monitoring could be useful in patients with long-QT syndrome as a low arousal positive affect (calm and relaxed) could be associated with QTc lengthening^21^.

Therefore, many different clinical conditions may require tight monitoring of the QT interval when an ECG might be not always available. Additionally, the possibility to perform remote monitoring of QT interval could be useful during periods of limited hospital resources and the need to avoid frequent in-person patient contact, such as during a pandemic.

Another advantage of QT measurement with a smartwatch is the possibility of zooming the pdf tracing, allowing for a more precise definition of the T wave end.

### Limitations of the study

There are several limitations to the present study that should be acknowledged. First, the smartwatch recorded the ECG trace in pdf format into a specific health application of the iPhone, which is necessary to print or send the ECG. Second, the measurement of the QT interval and the correct QT interval was done manually by the cardiologist. In the future, it might be desirable to obtain a system of automatic analysis, with or without the use of artificial intelligence, for an immediate warning in the presence of a pathological QT interval. Third, QT interval measurement was made only in patients in sinus rhythm. Further studies should be performed to assess the reliability of this device in patients with atrial fibrillation. Fourth, the recording of lead II and the V2 lead was made with assistance from medical personnel. For at-home smartwatch use, it is necessary to train the subject to accurately position the smartwatch on the body to obtain these two additional leads. In this setting, it could be helpful to obtain in the first instance the measurement of the QT interval in lead I, performing additional leads only in the case of difficulty with calculating the QT interval in this lead due to alterations in the T wave. Fifth, the study included relatively few patients (5 of 119) with obesity. Body habits or skin conditions may influence the image quality by affecting the QT interval’s measurement.

It should be pointed out that the mean age of our study population was 55 ± 23 years. The results of this study should be further validated in a larger number of subjects including more normal healthy controls.

We acknowledge that the Apple Watch does not have FDA approval for the modified leads that we used in the present study.

## Conclusion

This study demonstrates the feasibility of measuring QT and QTc with a commercially available smartwatch with results comparable to those measured with a standard ECG. These results could have an important clinical impact when frequent QT interval monitoring is required, especially for remote monitoring of QT interval when drug therapies that could affect the QT interval are required and access to hospital care may be limited, such as during a pandemic.

## Supplementary Information

Supplementary Information.
