# Supplementary material for: Measurement of the QT interval using the Apple Watch
Source: Sci Rep. 2021 May 24;11:10817. doi: 10.1038/s41598-021-89199-z (PMC8144193; doi:10.1038/s41598-021-89199-z)
Supplement: Supplementary file 1 — Supplementary Information. [file 41598_2021_89199_MOESM1_ESM.docx]

Supplementary figure 1

Figure S1

**Comparison of QT measured with the standard ECG and the smartwatch ECG (without stemi patient)**

Bland–Altman plot indicating the level of agreement between the smartwatch ECG and the standard 12-lead ECG measurement of the QT (ms) interval in lead I (panel A), lead II (panel B), and the V2 lead (panel C). The solid red line represents the bias and dashed red lines the upper and lower limit of agreement (LOA).


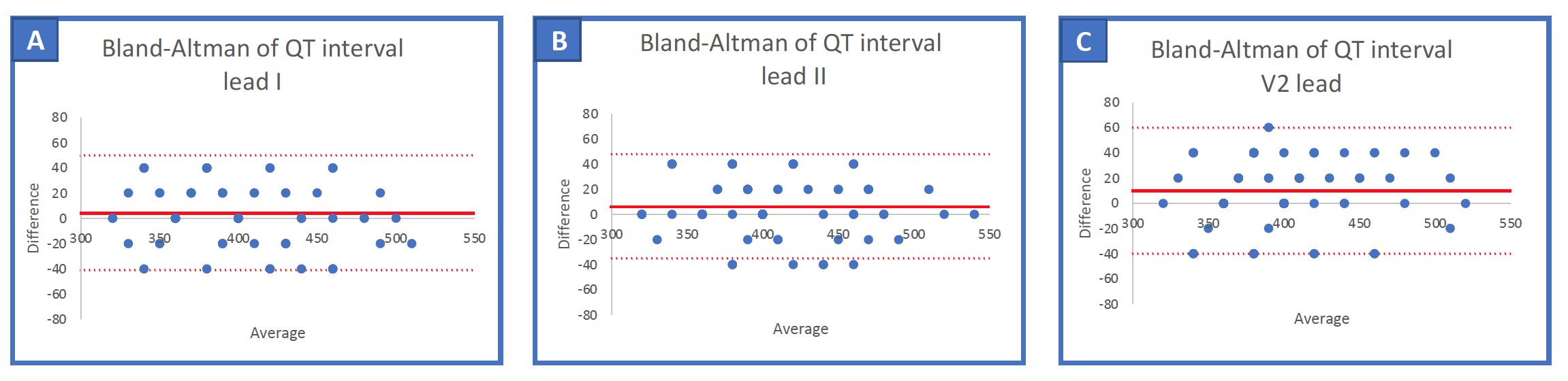


Supplementary figure 2

Figure S2

**Correlation of heart rate (HR)-a (ms) and HR-e (ms)**

Scatterplot and fitted line showing the linear association between the HR (measured as an average of three RR distances) performed using the smartwatch (HR-a) and standard 12-Lead ECG (HR-e).


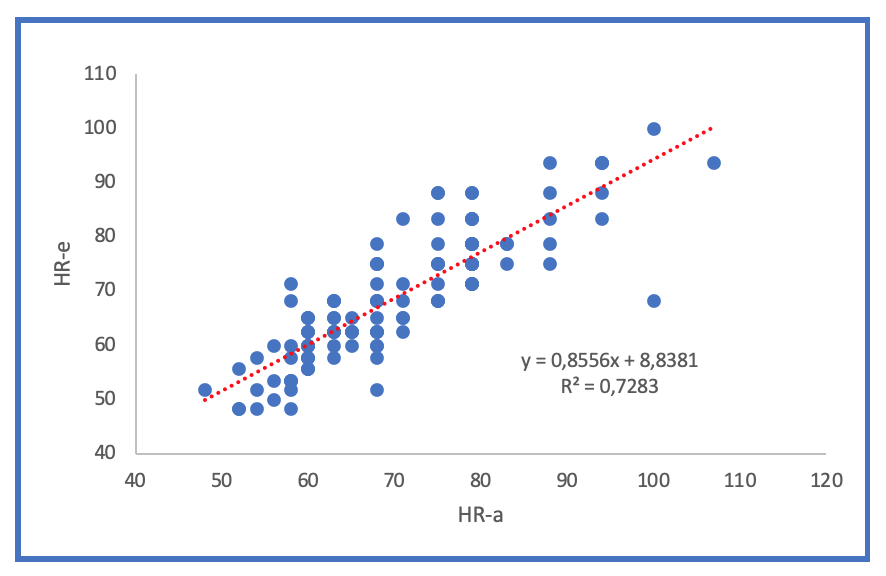


Supplementary figure 3

Figure S3

**Correlation of QT in-terval Lead I-a (ms) and QTLI-e (ms)**

Scatterplot and fitted line showing the linear association between the QT interval (measured in Lead I) performed using the smartwatch (QTLI-a) and the standard 12-lead ECG (QTLI-e).


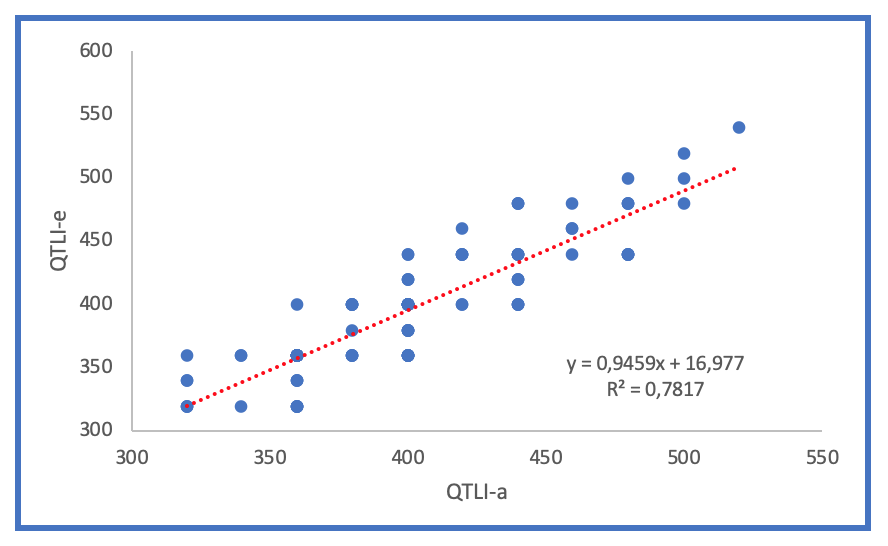


Supplementary figure 4

Figure S4

**Correlation of QT interval LII-a (ms) and QTLII-e (ms)**

Scatterplot and fitted line showing the linear association between the QT interval (measured in Lead II) performed using the smartwatch (QTLII-a) and the standard 12-lead ECG (QTLII-e).


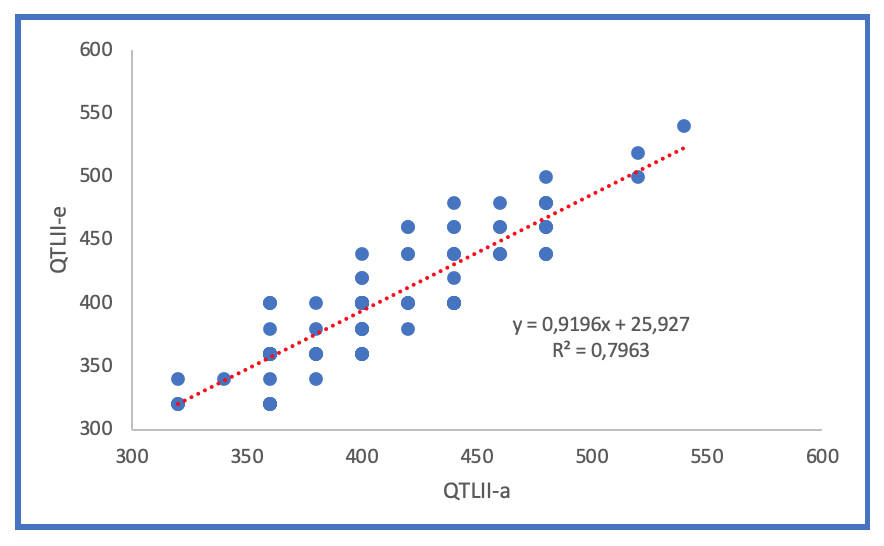


Supplementary figure 5

Figure S5

**Correlation of QT interval V2-a (ms) and QTV2-e (ms)**

Scatterplot and fitted line showing the linear association between the QT interval (measured in Lead V2) performed using a smartwatch (QTV2-a) and the standard 12-lead ECG (QTV2-e).


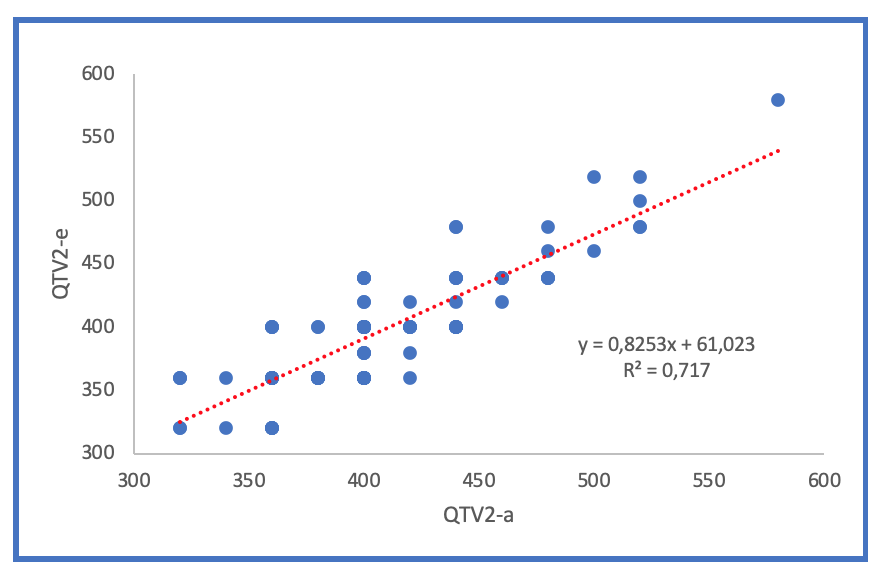


Supplementary figure 6

Figure S6

**A representative ex-ample of prolonged QT interval detected by standard 12-lead ECG (Panel A) and by the smart-watch (Panel B) in the same patient**

In panel A, the figure shows LI, LII, and V2 leads performed using a standard ECG and panel B shows LI, LII, and the V2 lead obtained with the smartwatch ECG


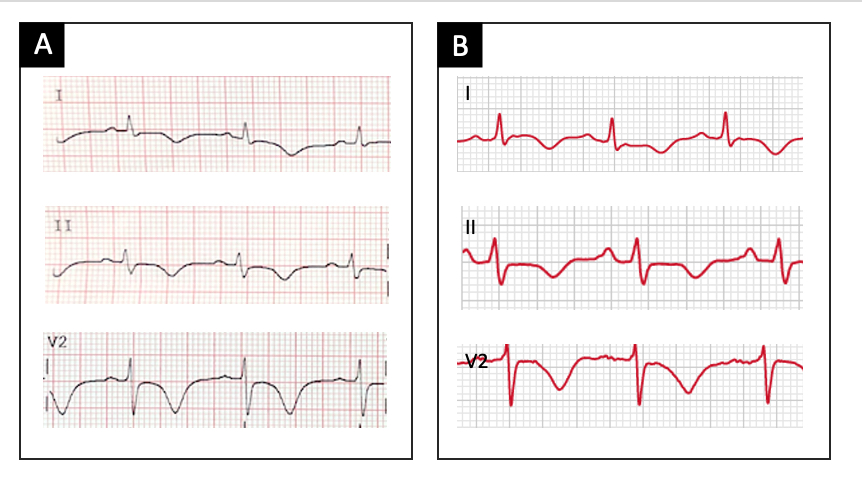


Supplementary figure 7

**Comparison of QTc measured with the standard ECG and the smartwatch ECG (without stemi patient)**

Bland–Altman plot indicating the level of agreement between the smartwatch ECG and the standard 12-lead ECG measurement of the QTc (ms) interval in lead I (panel A), lead II (panel B), and the V2 lead (panel C). The solid red line represents the bias and dashed red lines the upper and lower limit of agreement (LOA).


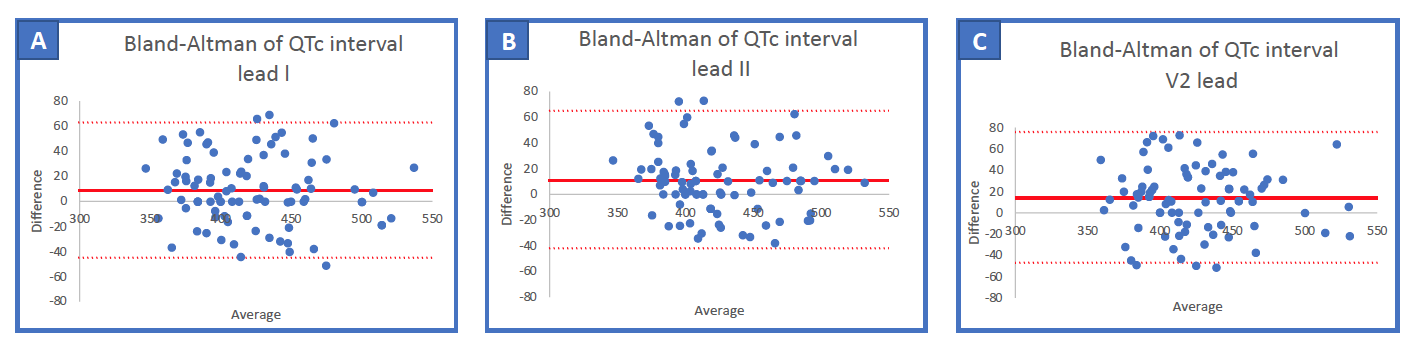


Supplementary Table 1

**QT interval of average of each lead ( LeadI-LeadII-V2lead) using standard ECG and apple ECG***.*

| QT mean  (ECG standard) | QT mean  (ECG apple) |
| --- | --- |
| 373 | 373 |
| 373 | 400 |
| 520 | 507 |
| 413 | 440 |
| 440 | 453 |
| 453 | 493 |
| 440 | 467 |
| 347 | 373 |
| 473 | 487 |
| 440 | 440 |
| 340 | 360 |
| 400 | 407 |
| 453 | 487 |
| 333 | 340 |
| 380 | 387 |
| 360 | 380 |
| 460 | 427 |
| 333 | 373 |
| 373 | 380 |
| 400 | 400 |
| 400 | 427 |
| 393 | 413 |
| 460 | 467 |
| 433 | 447 |
| 487 | 467 |
| 373 | 360 |
| 360 | 360 |
| 373 | 400 |
| 373 | 367 |
| 373 | 387 |
| 453 | 440 |
| 420 | 440 |
| 360 | 367 |
| 373 | 380 |
| 360 | 373 |
| 373 | 400 |
| 367 | 387 |
| 447 | 453 |
| 393 | 420 |
| 347 | 347 |
| 433 | 440 |
| 367 | 380 |
| 353 | 373 |
| 440 | 467 |
| 433 | 460 |
| 427 | 440 |
| 407 | 393 |
| 447 | 447 |
| 353 | 387 |
| 327 | 360 |
| 413 | 420 |
| 407 | 407 |
| 407 | 420 |
| 427 | 420 |
| 433 | 467 |
| 387 | 413 |
| 413 | 400 |
| 360 | 380 |
| 347 | 387 |
| 360 | 373 |
| 367 | 400 |
| 373 | 387 |
| 400 | 413 |
| 400 | 380 |
| 387 | 360 |
| 427 | 440 |
| 380 | 373 |
| 333 | 340 |
| 373 | 373 |
| 373 | 407 |
| 400 | 400 |
| 340 | 353 |
| 333 | 333 |
| 400 | 400 |
| 527 | 533 |
| 433 | 420 |
| 460 | 467 |
| 400 | 393 |
| 480 | 453 |
| 340 | 333 |
| 387 | 400 |
| 400 | 407 |
| 413 | 393 |
| 500 | 500 |
| 400 | 427 |
| 440 | 413 |
| 407 | 427 |
| 413 | 427 |
| 413 | 407 |
| 373 | 393 |
| 407 | 407 |
| 400 | 407 |
| 473 | 433 |
| 360 | 360 |
| 347 | 333 |
| 373 | 407 |
| 413 | 400 |
| 453 | 427 |
| 433 | 413 |
| 493 | 500 |
| 360 | 360 |
| 380 | 387 |
| 373 | 373 |
| 373 | 380 |
| 400 | 400 |
| 373 | 387 |
| 373 | 373 |
| 373 | 380 |
| 360 | 373 |
| 367 | 373 |
| 320 | 320 |
| 387 | 400 |
| 360 | 367 |
| 400 | 400 |
| 400 | 400 |
| 360 | 360 |
| 333 | 347 |
| 387 | 400 |
| 373 | 373 |
